# Promoting resident thriving in nursing homes: A qualitative study

**DOI:** 10.1111/jan.16206

**Published:** 2024-04-25

**Authors:** Rebecca Baxter, Laura Corneliusson, Sabine Björk, David Edvardsson

**Affiliations:** ^1^ Department of Nursing Umeå University Umeå Sweden; ^2^ Institute of Health and Care Sciences, Sahlgrenska Academy University of Gothenburg Gothenburg Sweden; ^3^ School of Nursing and Midwifery La Trobe University Melbourne Victoria Australia

## Abstract

**Aim:**

To explore how staff promote resident thriving in an Australian nursing home.

**Design:**

Qualitative research design using content analysis.

**Methods:**

Interviews were held with 14 nursing staff working in an Australian nursing home in March/April 2018. Data were analysed using qualitative content analysis.

**Results:**

Four themes were revealed: promoting personalized care; promoting opportunities for autonomy; promoting connection and meaning; and promoting a curated environment.

**Conclusions:**

Staff promoted resident thriving in relation to everyday care, activities, capabilities, relationships and the lived environment. Interventions that were perceived to promote thriving were described relative to the nurse, the resident, the care team and the wider nursing home context.

**Implications for the Profession and/or Patient Care:**

The findings provide novel insight into the understanding and application of the concept of thriving in long‐term care from nursing staffs' perspective.

**Impact:**

**What Problem did the Study Address?**

Nursing home staff are uniquely positioned to support resident thriving through person‐centred care, yet little is known about how staff support promotion of thriving for nursing home residents in their everyday practice.

**What were the Main Findings?**

Nursing home staff perceived that they promoted thriving by personalizing care and supporting autonomy, connection, meaning and pleasure for residents.

**Where and on Whom will the Research have an Impact?**

These findings are of interest for the clinicians, educators and researchers who develop, implement and evaluate interventions to improve place‐related well‐being in nursing homes.

**Reporting Method:**

CORE‐Q checklist.

**Patient or Public Contribution:**

No patient or public contribution occurred for this study as this research focused on exploring staffs' perspectives from the specific viewpoint of their personal experience.

## 1 INTRODUCTION

Thriving is described as resulting from optimal interactions between a person and their human and non‐human environment, indicating a good fit between the person and their life‐world (Haight et al., 2002). In the nursing home context, residents who thrive are said to experience place‐related well‐being as they have settled into the nursing home and perceive their life to be as good as it can be, despite potential physical or cognitive decline (Bergland & Kirkevold, 2001; Bergland & Kirkevold, 2006). In this way, thriving in nursing homes can be understood as a holistic and health‐promoting concept that denotes lived experiences of situated contentment in relation to personal, social, spacial and societal frameworks (Baxter, Corneliusson, et al., 2021). Thriving in nursing homes is multifaceted and reflects the quality of interactions between residents and their environment. By creating an environment that is conducive to residents' place‐related well‐being, nursing homes could support a sense of situated contentment that could possibly mitigate some of the negative experiences related to decline in later life (Baxter, Corneliusson, et al., 2021). It would therefore appear that staff in nursing homes are in a unique position to enable and improve thriving through targeted care interventions relative to their nursing knowledge, their interactions with the resident and their role in the lived environment. Yet, research is lacking regarding how nurses view their role in promoting resident in nursing homes, and little is known about the specific interventions that staff undertake to promote thriving.

## 2 BACKGROUND

Australians live in an ageing society and the number of older adults is expected to more than double in the next 30 years (Australian Institute of Health and Welfare Older People, 2022). In the last decade, the proportion of Australians living in permanent residential aged care increased by 15% and demand for such services is predicted to increase as the likelihood of multi‐morbidity and physical or cognitive impairment rises with advancing age (Australian Institute of Health and Welfare Older Australians, 2022). There has been a clear impetus to move towards more progressive care models in nursing homes to better cater to the full spectrum of resident needs (Theurer et al., 2015). Past research has shown a strong association between quality nursing care and resident well‐being in nursing homes (Cleland et al., 2021). Maintaining the health and well‐being of this population group therefore ought to be at the forefront of education, policy and practice, yet there is little in the way of health‐promotion for older people living in nursing homes. The concept of thriving shows promise in this regard as it encompasses positive experiences of care, support, relationships and the physical environment.

Bergland and Kirkevold described several core aspects for thriving in Norwegian nursing homes, namely the residents' own attitude towards living in the nursing home and the quality of care and caregivers (Bergland & Kirkevold, 2006). Other contributing aspects included the physical nursing home environment, opportunities for activities and relationships with peers and family. For residents with dementia, thriving and positive subjective experiences were said to be supported through contentment, acceptance, support and relationships (Mjørud et al., 2017). Residents in an Australian nursing home described meanings of thriving as having options, choices and a sense of agency to influence their everyday care and environment (Baxter, Sandman, et al., 2019). Experiences of thriving involved balancing daily care provision and creating a sense of agency for residents, along with creating a welcoming physical environment and fostering positive relationships with caregivers and peers (Baxter, Sandman, et al., 2019). American nursing home residents also highlighted the importance of engagement, a positive attitude, reflection, decision‐making and adaptation as being central to thriving (Sullivan & Willis, 2018). While cultural or contextual differences may exist, these resident descriptions of thriving appear to be less related to individual *abilities* and are instead more closely linked to personal and environmental *possibilities*. Importantly, experiences of resident thriving are described in spite of declining physical or cognitive status (Bergland & Kirkevold, 2006). Resident descriptions indicate that support from staff can play a role in their overall experience of thriving, which is why it is important to explore the extent to which resident experiences align with staff perceptions of thriving‐promoting interventions in praxis.

Australian nursing home staff demonstrated sound understanding of the concept of thriving, describing recognition of expressions of thriving among residents as occurring though a process of understanding, observing and sensing (Baxter, Sandman, et al., 2021). Staff articulated that they used both professional assessment skills and their personal intuition based on their knowledge of the resident as a whole person to determine whether residents were thriving or not (Baxter, Sandman, et al., 2021). American nursing home staff likewise viewed resident thriving as fluid and dynamic, with the role of the nurse described as pivotal in relation to providing encouragement, emotional support, medical help, appropriate activities, safety and person‐centred care (Sullivan & Willis, 2018). Comparisons of resident and staff descriptions of thriving reflected that staff may view resident health as playing a larger role in thriving than the residents themselves described (Sullivan & Willis, 2018). While staff and residents both indicate that the possibility to thrive could be impacted by the amount of support provided in the lived environment (Baxter, Sandman, et al., 2019, 2021; Sullivan & Willis, 2018), little is known about how staff undertake thriving promoting interventions in their daily interactions with residents.

## 3 THE STUDY

### 3.1 Aim

This study aimed to explore how staff promote resident thriving in an Australian nursing home. The research question was: How do staff describe promoting resident thriving in the nursing home?

## 4 METHODS

### 4.1 Design and theoretical framework

A qualitative descriptive design underpinned by phenomenological hermeneutics facilitated qualitative content analysis of experiential data. A phenomenological hermeneutical approach seeks to comprehend phenomena from the perspective of those experiencing it and refers to both understanding and interpretation (Lindseth & Norberg, 2022). This supports a nuanced understanding of participants' subjective experiences and how the meanings of these encounters are shaped by a person's internal and external lifeworld. By using phenomenological hermeneutics as the theoretical framework it becomes possible to uncover deeper meanings in the function and character of participants' everyday experiences. This study could therefore provide insight into how staff in an Australian nursing home promote thriving among residents, which may add nuance to cross‐cultural and global understandings of the phenomenon of thriving.

### 4.2 Study setting

The Department of Health and Aged Care (Department of Health and Aged Care, 2024) outlines that any Australian aged 65 years or older (or 50 years or older for persons who identify as an Aboriginal or Torres Strait Islander) may access government funded aged care services. Residential aged care homes (nursing homes) are available for older people who are unable to live at home safely, who need ongoing assistance with activities of daily living or who require health care (Department of Health and Aged Care, 2024). Australian nursing homes can be public or private and offer different levels of care depending on individual needs. The cost of care and accommodation varies based on means‐testing and government subsidies. Minimum staffing requirements are determined based on the number of residents and the level of care they require. Nursing homes are usually staffed by registered nurses, enrolled nurses and care assistants.

This study was undertaken at a nursing home in rural Victoria, Australia, that provided residential aged care, palliative care, respite care and secure dementia care. The nursing home was located close to the local hospital and township and contained common areas that were used for activities, relaxation, meetings or functions. Residents lived in single or double rooms with shared or private bathrooms. Care requirements varied depending on the reason for admission. A registered nurse was always on‐site.

### 4.3 Participants and recruitment

Purposive recruitment of participants commenced in March 2018. The nursing home manager granted permission for the first and last authors to present the study objectives at the monthly staff meeting. If staff were interested in participating, they were asked to contact the nursing home manager who booked an interview time. Staff were eligible for interview if they were aged 18 years or older, had worked at the nursing home for at least 3 months, held a qualification as a Registered Nurse or Enrolled Nurse and were able and willing to provide informed consent.

### 4.4 Data collection

The interviews took place between March and April 2018. The first two interviews were conducted with the first (Female, PhD candidate, Registered Nurse) and last (Male, PhD, Professor, Registered Nurse) authors, and all remaining interviews were conducted by the first author. Both interviewers were familiar with thriving theory and had experience in clinical and academic nursing arenas. All interviews took place in a private meeting room in the nursing home and were negotiated around the participants' daily work schedule. To develop a common understanding of the concept of interest, participants were first asked to describe thriving (e.g., Could you describe what thriving means to you?). Participants were then asked how they promoted thriving in their everyday clinical practice at the nursing home (e.g., What could/would you do to promote resident thriving). Follow up questions were guided by participant responses. The interviews lasted between 18 and 41 min and audio recordings were transcribed by the first author for analysis. After 14 interviews no additional information materialized and we reached what we considered to be saturation (Saunders et al., 2018).

### 4.5 Data analysis

Qualitative content analysis was conducted using Graneheim and Lundman's (Graneheim et al., 2017; Graneheim & Lundman, 2004) framework. An inductive approach was selected, meaning that thematic construction emerged from the data as no initial hypothesis was used to guide the preliminary coding and subsequent thematic development (Graneheim et al., 2017). First, the interview texts were read in their entirety to establish an overall understanding of the content. The first author divided the text into meaning units, that is, sections of text that conveyed a single meaning related to the study aim. As shown in Table 1, meaning units were condensed, coded and eventually grouped in relation to overall content area. The groupings formed the basis for the interpretation and development of emergent subthemes. Finally, four themes that unified the subtheme content were formulated and compared with the original text to confirm their application and relevance. All authors agreed on the final results.

**TABLE 1:** Example of analysis process.

Meaning unit	Condensed meaning unit	Interpretation	Sub‐theme	Theme
‘Every second day here they choose their own meals, they are not the same. We leave fruit at the table in the common area so that they can have fruit at any time they like. If you want to have a wine, we encourage that. For them to have that choice, it's a good thing. It's a small part, but it makes them happy’ (N12)	Residents can choose their own meals. They can choose to have fruit any time they like. They can have a wine if they want to. Having choices is a good thing. It makes them happy.	Giving residents options and opportunities to make choices makes them happy.	Respecting preferences	Promoting opportunities for autonomy

### 4.6 Ethical considerations

Ethical approval for the study was granted by the La Trobe University Human Research Ethics Committee (S17‐228). The researchers had no pre‐existing relationships with the nursing home staff or residents. Information statements about the study were provided in advance of the interviews, and written consent was obtained from all participants. Reasons for non‐participation were not explored. To protect participant anonymity, the supporting quotation sources are referred to as ‘N’ for ‘Nurse’ with an identifying number (e.g., 1–14).

### 4.7 Rigour and reflexivity

To ensure rigour, the COREQ checklist criteria were addressed in the reporting of the study. The authors actively engaged in reflexivity and discussion throughout the analysis process. This included reflection on the researchers' clinical/professional expertise, specifically as Registered Nurses (RB, SB, DE) and Social Workers (LC), as well as any thoughts or feelings that emerged during data analysis. All authors played a significant role in the data analysis and development of the final themes. A transparent description of all steps taken in conducting this study has been provided, as well as citation of methodological papers that describe data analysis processes in detail. Risk of bias was reduced by bracketing and using an iterative approach to data analysis, revisiting the data multiple times and framing the subthemes using language that remained close to the original data. Confirmability was considered though the provision of detailed descriptions of the various categories and supporting quotations. Transferability was addressed by clearly describing the study design, setting and research process should other studies wish to replicate these methods in the future.

## 5 FINDINGS

Fourteen nursing staff were interviewed (2 Registered Nurses; 12 Enrolled Nurses). The participants had a mean age of 46.6 years, were mostly female (*n* = 12), and their number of years of nursing experience ranged from 3 to 40 years (mean, 21.7 years).

The analysis revealed four themes and nine subthemes to illustrate how staff promoted thriving for nursing home residents (Table 2). The themes were: promoting personalized care (three subthemes); promoting opportunities for autonomy (two subthemes); promoting meaning and connection (two subthemes); and promoting a curated environment (two subthemes). The themes are outlined with an initial description, followed by details of their subthemes and supporting quotations (in italics).

**TABLE 2:** Summary of themes.

Theme	Subtheme
Promoting personalized care	Knowing the person Working as a team Doing the essentials and the extras
Promoting opportunities for autonomy	Encouraging capabilities Respecting preferences
Promoting meaning and connection	Building and bridging relationships Balancing variety and pleasure
Promoting a curated environment	Making the inside enjoyable and functional Making the outside beautiful and practical

### 5.1 Promoting personalized care

Basic everyday care provision was described as necessary to support resident thriving, but staff described going beyond the essential requirements by developing personalized knowledge of residents' preferences and adopting a team approach to care provision. Promoting personalized care comprised of three subthemes: knowing the person, working as a team and doing the essentials and the extras.

#### 5.1.1 Knowing the person

Participants described the importance of knowing the person to promote thriving in their everyday care. Staff attempted to ‘*build a rapport with them*’ (N13) and ‘*try to get them to open up*’ (N14) by talking to residents about their likes, dislikes and history because ‘*you've got to remember that these are people who have got their own stories and their own lives – we allow that to shine through*’ (N7). This allowed nursing staff to get to know the person on a deeper level so that they were able to perceive and anticipate their needs. As one staff member recounted, ‘*you know certain things about people, they want things a certain way. Some will want an additional drink at a certain time of day. One (resident) here will have his wine at 4pm so automatically you can get that so then the person doesn't become anxious, they don't have to wait, and they don't have to ask for it – they know automatically that you will have that there and that is appreciated by them*’ (N6). Taking the time to learn about residents was perceived to make them feel seen, understood and safe. Staff said that the process of getting to know residents involved sharing, reciprocity and occurred over time, but to begin this process they could simply ‘*just sit down and have a conversation, ask them about their life*’ (N2) and ‘*go and sit and listen to these people's stories, and share your stories ‐ they love hearing our stories too*’ (N9).

#### 5.1.2 Working as a team

Teamwork among staff was described as vital to supporting thriving for residents through thoughtful and consistent care delivery. If a staff member identified that a resident was not thriving they indicated that they would discuss it at handover because, ‘*we all have to be on the one page, on the same team*’ (N4). From there, it was possible to come up with strategies and coordinate efforts, ‘*we will say such and such (resident) is feeling a bit flat, this may have happened, you know. Then you can go in and assess that person your way*. *We all come at it from different angles*’ (N13). This involved both formal and informal communication among the care team, as well as documentation in the clinical notes and at handover about particular care strategies that did (or did not) work to promote thriving for individual residents because, ‘*we are all adaptable, we all want to learn*’ (N11). As one staff member narrated, ‘*usually, we discuss them at handover and do something about it. We would say, ‘you have got to work that through’. You work out what is. We discuss it… it's like all things ‐ encouragement that has worked for someone, we discuss that so it keeps happening*’ (N2).

#### 5.1.3 Doing the essentials and the extras

Provision of high‐quality everyday nursing care was described as a foundation for resident thriving. These essential cares were discussed in relation to supporting activities of daily living, such as showering and dressing, and in terms of more specialized responsibilities, such as treatment and rehabilitation. One staff member expressed, ‘*the quality of care given here is exceptional, on the whole it is just terrific. We have skin integrity, it tells a picture. Yes, we have chronic wounds, but we don't have nasty pressure sores, you know, because the patients are turned and they aren't neglected… So I think that the care here does help them thrive and flourish if they want to*’ (N1). Staff felt a sense of accountability surrounding resident success in relation to the care that they provided, saying ‘*I have to think ‘what can I do for these patients’? …It all comes back to us*’ (N12). Tasks were viewed as somewhat hierarchical, with one staff member outlining, ‘*hygiene is basically at the top, then the medications, and then it all just flows together*’ (N3). However, staff also described that there was a difference between simply doing essential tasks and going beyond to do the ‘extras’. Staff described that it was important to ‘*focus on bowels, tablets, the main things. They are the bare essentials. But the extras, that comes from the regular staff. If you know the small things, something that people wouldn't always think of, you know ‐ that they like that pink lipstick, stuff like that*’ (N11). Doing the essentials could provide opportunities to ‘*go the extra mile*’ (N7) and ‘*do the little things*’ (N2) as the ‘extras’ often occurred parallel to basic care provision to promote thriving. As one staff member reflected, ‘*I am a bit of a slowpoke with my hygiene because I take a little bit longer, but I try and spend a little bit more time and give them more… you know, when I wash their hair I will spend some time and give them a massage, those little extra things, instead of rushing them*’ (N7).

### 5.2 Promoting opportunities for autonomy

Promotion of thriving involved ensuring that residents had a sense of autonomy and self‐determination in the nursing home. Staff strived to achieve this by providing access to things that the resident liked and through offering options and choices across all levels of care. Promoting opportunities for autonomy comprised of two subthemes: encouraging capabilities and respecting preferences.

#### 5.2.1 Encouraging capabilities

Participants described the importance of encouraging resident capabilities when promoting thriving. This involved using positive reinforcement, encouragement and praise when a resident successfully completed a task or activity, particularly if they lacked confidence with that task previously. Staff were conscious to find a balance between doing things for residents and supporting them to do things for themselves, ‘*we like to encourage that independence, but we are always there to assist them*’ (N13). Participants described using ‘*kind gentle nudges*’ (N13) to encourage residents to build confidence in their abilities. They acknowledged that residents could lose their sense of independence when moving to a nursing home, so encouraging them to feel secure in their (adjusted) capabilities again was important. One staff member articulated, ‘*if you don't use it, you lose it! So, we try and encourage them the whole time to be involved, to actually walk, to do this, and to do that ‐ so they actually get out of their little rut and keep going. A lot of it is praise. You have to really encourage them and praise them*’ (N2). In this way, staff attempted to shift residents' focus away from that which they could not do, towards the things that they could do, ‘*we are always there, but we encourage them to do as much as they possibly can*’ (N13).

#### 5.2.2 Respecting preferences

The connection between thriving and respecting resident preferences was acknowledged by providing opportunities for residents to do what they wanted, when they wanted and how they wanted in relation to all aspects of life in the nursing home. Staff embodied this by consciously asking the resident what they wanted to eat, wear and do on a daily basis, and by continuing to check in with residents at different times (e.g., weeks, months and years) to assess if their desires had changed, ‘*yeah, just asking when they come in, and throughout their stages, what they like to do ‐ and we document what they like to do and what upsets them and what stresses them*’ (N3). It was acknowledged that residents did not all have the same wants or needs and that these differences should be respected, for example, in relation to socializing, ‘*there are some people who are very content in their own company just doing their own thing… They don't want to mix with other people, or not as much as the others do. But there are some people who have that preference, and you have to appreciate and accept that*’ (N3). However, staff did not always agree with resident choices, particularly if the staff member thought it could have a negative impact on the resident's health, such as if a person did not want to get out of bed for days on end. In instances such as these, staff endeavoured to show residents respect by offering alternatives, such as helping the resident to change positions or assisting them to sit out in a chair; but ultimately the resident could make the final decision. Situations such as these were characterized by one nurse using the metaphor, ‘*you can only sow the seed, but you can't make people do anything, you know?*’ (N10).

### 5.3 Promoting meaning and connection

Meaning and connection to promote thriving were described as occurring through provision of interesting and pleasurable activities to do during the day. This also involved staff acting as a conduit for relationships with staff, other residents, family or other people. Promoting meaning and connection comprised of two subthemes: building and bridging relationships, and balancing variety and pleasure.

#### 5.3.1 Building and bridging relationships

Participants emphasized the importance of supporting resident relationships with staff, family and friends to enhance thriving because ‘*they are their own little community*’ (N8). Relationships with other residents were viewed as part of the wider social environment, and staff supported residents to get to know one another and connect during everyday encounters, ‘*we try and get them to interact with the other residents and take part in the activities and become a part of the home environment. That's what we get them to do so that they can thrive in their home*’ (N7). For some residents, maintaining connections with family, friends and other people outside the nursing home was significant and, if possible, nursing staff worked with residents' loved ones to provide better care. One participant recounted incorporating family knowledge with their own care approach in trying to encourage a resident to gain weight following an illness, ‘*I asked the family to bring in some foods that she liked to eat at home. She likes cheese, so the family brought in some. You have to put the family in there because they know them better than us*’ (N12). Relationships between staff and residents varied depending on the needs and wants of the person, encompassing a caring and professional side, as well as a closer personal side, ‘*we listen to them and they can sort of become like family because you are working with the same people every day… They look on you as part of their family because for some of them we're all that they've got*’ (N7).

#### 5.3.2 Balancing variety and pleasure

Offering a range of pleasurable activities to do during the day required shared knowledge about what residents enjoyed so that staff could plan activities that residents would find interesting, stimulating and fun. Staff described facilitating a variety of activities to ‘*make them happy*’ (N14) and ‘*have some fun with them and make them smile*’ (N9), such as reading the newspaper in a group, playing bingo, listening to music, watching movies, exercise classes, special resident lunches, hosting public figures, watching sports and going on daytrips. Residents were also welcome to participate in everyday activities within the nursing home, such as gardening and food preparation, ‘*they have independence, but don't have to worry about the small things. There is heaps of fun stuff to do… and they've got more time to spend on that stuff. They still get to cook, they have the classes and all of that stuff, but it doesn't have to be their main focus*’ (N11). When developing activities staff described that their thought process included consideration of how to best meet the needs of residents on both individual and collective levels in an attempt to fulfil their social and emotional needs. This was articulated by one staff member who stated, ‘*that is where you try and do things that will help them to thrive. Like, it could be something as little as finding out what their favourite types of movies are and going to the DVD library and seeing if there are some of those movies. Little things that can engage them you know. Giving them some joy, some attention you know, something to focus on*’ (N7).

### 5.4 Promoting a curated environment

Thoughtful organization and presentation of the lived nursing home environment was recognized by staff as impactful to promotion of thriving. The environment was spoken of in terms of the indoors and the outdoors, with unique features of both settings thought to contribute to thriving, both literally and metaphorically. Promoting a curated environment comprised of two subthemes: making the inside functional and enjoyable, and making the outside beautiful and practical.

#### 5.4.1 Making the inside functional and enjoyable

Staff indicated that the ‘inside’ of the nursing home could be adjusted and optimized to promote thriving. This was spoken of in terms of functionality for resident needs, including accessibility of hallways, safety of rooms and clean living spaces. Staff recalled that the lived environment had been changed many times based on resident feedback, ‘*we should be making it more of a home setting, and making it a fun setting*’ (N9). Function had to be balanced with making the nursing home enjoyable, welcoming and homely. It was acknowledged that some aspects of the nursing home were not able to be altered due to health and safety regulations (both for staff and residents), ‘*there is a genuine belief and attempt to make it like home. But at the end of it, just by sheer definition, it will always be an institution*’ (N5). Aspects of the nursing home that could be curated to residents' tastes were considered carefully. The walls of the nursing home displayed art from local artists and fresh plants were installed throughout the living areas. As one staff member narrated, ‘*it is a very clean environment, the rooms are lovely, they can have their own touches to it. You know, pictures, photos, furniture, things like that. They've got a lovely day room… They have a lot of input into their home, because this is their home*’ (N7).

#### 5.4.2 Making the outside beautiful and practical

The ‘outside’ environment was described as significant both in terms of aesthetics and practicality to support thriving. Staff were involved in the design of the garden, describing that thought went into every aspect to make the outdoor spaces enjoyable and interactive for residents. Residents were supported to be outside by staff in practical ways, such as leaving the doors unlocked to the internal courtyard and providing appropriate, comfortable seating with wide paths to accommodate mobility aids. It was important for the garden to be accessible because the staff were ‘*always trying to promote them to get outside and get fresh air*’ (N3). Activities such as gardening could be incorporated with resident's rehabilitation, and aesthetically appealing plants could be interacted with in terms of their colours, scents and care, ‘*even when they are out there watering the garden in the heat ‐ you cringe, but you enjoy them getting involved*’ (N4). The outdoors offered both literal and metaphorical opportunities for the resident to change their lived environment on their terms, ‘*once you come out that door, things can open up to you… planting that seed is important. In the garden, and in the mind*’ (N4).

## 6 DISCUSSION

This study revealed four themes and nine subthemes to describe how nursing staff promoted thriving in everyday care. Nursing home staff promoted thriving by personalizing resident care and supporting autonomy, connection, meaning and pleasure. Seeking both general and specific information about a person has been said to be crucial to being able to individualize care around things that are important to the person, such as their life history, significant relationships, and likes and dislikes (Ho et al., 2021). This is consistent with research regarding person‐centred care in nursing homes, where staff prepared for their role as a carer by actively seeking out information about resident needs and expectations (Hedman et al., 2022). This study also highlighted that information gathering and sharing extended beyond individual nursing staff to the wider multidisciplinary context, as what was shared with one staff member could be disseminated among the wider care team though face‐to‐face communication or through documentation in the clinical notes. These aspects culminated in nursing staffs' ability to provide care that encompassed both *the essentials* and *the extras* to promote thriving. Doing more than what was typically expected has been expressed by nursing home staff as characteristic of working in a person‐centred way (Vassbø et al., 2019). Residents too have expressed that feeling seen, understood, supported and cared for are central to the meanings of thriving (Baxter, Sandman, et al., 2019). Research regarding person‐centred care in nursing homes has largely focused on delineating conceptual content, policy directions and management strategies, with less focus directed towards exploring how nursing staff enact such concepts in practice (Edvardsson et al., 2014). Explicating facets of thriving‐promoting care could therefore align resident experiences with staff knowledge and skills through shared contextual, interactional and experiential understandings.

Staff emphasized that the nursing home could reveal new or adapted opportunities to promote resident thriving through a capabilities‐focused approach, for example by providing residents with encouragement, reassurance or a sense of security. This emphasis on interactional inspiration, respect and encouragement supports the findings of previous research that showed bodily indicators were less meaningful than existential and experiential aspects of thriving among nursing home residents (Baxter, Björk, & Edvardsson, 2019; Baxter, Sandman, et al., 2019; Sullivan & Willis, 2018). Indeed, fostering a sense of autonomy though supportive participation has been shown to facilitate feelings of power, independence and choice (Santos et al., 2008). Thriving therefore seems to show promise as a health‐promoting care framework through which capabilities and strengths‐focused interventions can be conceptualized, understood and embodied in everyday nursing home care. Further, as it is possible to measure thriving using a validated scale, interventions to support thriving could be quantified for auditing and quality improvement purposes in future research (Baxter, Lövheim, et al., 2019; Bergland et al., 2015).

Promoting meaning and connection through relationships, variety and pleasure compliments research reporting positive associations between thriving and inclusion in everyday events, structured activities, dressing well and spending time with enjoyable people (Björk et al., 2017). Feelings of thriving have also been said to emerge through connections with loved ones, communication with care providers, a pleasant care environment, feeling secure and respected, and being satisfied with life (Ericson‐Lidman, 2019). Several studies noted that aggression, depression and cognitive impairment were associated with lower thriving (Baxter et al., 2022; Björk, Lövheim, et al., 2018) and quality of life (Patomella et al., 2016). Given the major issues in long‐term care that were exposed as a result of the COVID‐19 pandemic (Edvardsson et al., 2020) it seems important to explore how positive experiences and outcomes for residents with physical, cognitive, functional or social concerns can be supported during periods of isolation or distance. Moreover, the pandemic exemplified that although staff are well positioned to implement complex care interventions, structural and organizational support is required to sustain high‐quality care delivery. In this way, continued exploration of concepts such as thriving from resident, staff and organizational perspectives provides opportunities for development of evidence‐based and meaningful care interventions that are more pre‐emptive than reactive.

Staff expressed that the internal/external places and spaces of the nursing home could have a substantial impact on resident thriving. The contrast of the nursing home as both a home and an institution has been described by both residents and staff as an important factor for thriving (Baxter, Corneliusson, et al., 2021; Baxter, Sandman, et al., 2019, 2021), with perceptions of homeliness said to be influenced by psychological, social and built factors (Rijnaard et al., 2016). Prior research found that internal environmental features such as a positive psychosocial climate and having access to the wider community through newspapers and not locking doors to the nursing home were associated with higher levels of thriving (Björk, Lindkvist, et al., 2018). Likewise, the natural outdoor environment has been said to support well‐being and quality of life, as well as generating positive aesthetic, sensory and emotional responses (Orr et al., 2016). Outdoor surroundings have been posited as important therapeutic environments, offering opportunities for residents to engage, reminisce and share their experiences and memories in nature (Johansen & Gonzalez, 2018). Making the indoor and outdoor environments homely, inviting, safe and user‐friendly provides insight into how staff can intervene to create and curate residents' lived environment.

Staff perceived that they had an important role in supporting thriving, illuminated as a commitment and willingness to facilitate resident thriving through direct and indirect interventions. Sjögren et al. explored perceptions of quality of care after staff underwent an education programme on person‐centred care and thriving, finding that staff education had a positive impact on (male) residents' experience of thriving (Sjögren et al., 2022). The education programme protocol included features such as the staff doing ‘a little extra’, exemplifying the role of the staff as facilitators for thriving. It is possible that resident thriving may be supported by continued staff education, but such interventions require resources in the form of time and money. These findings therefore may be useful for clinicians, managers and organizations in allocating and prioritizing resources or interventions that are described by both residents and staff as important for thriving promotion. The actions described herein may also be useful in the development of clinical practice guidelines to better monitor and enhance thriving in nursing homes.

### 6.1 Strengths and limitations

The qualitative research design allowed for a novel exploration of how nurses described supporting resident thriving in nursing homes. Participants were purposively recruited which risks self‐selection bias; however, this sampling method can also be viewed as a strength as participants were willing and able to talk about the subject matter. The study was undertaken in a single nursing home in rural Victoria, Australia, and as a result, we acknowledge that the experiences may differ from that of staff working in other care settings or contexts. For this reason, we have described the overall Australian residential care setting (Department of Health and Aged Care, 2024) and specific features of the nursing home in this study. In keeping with Graneheim and Lundman's (Graneheim et al., 2017; Graneheim & Lundman, 2004) qualitative content analysis methodology, it is left to the reader to determine if the findings from this study can be transferred to other contexts. The role of the researcher is pivotal in the qualitative research process, it is therefore important to acknowledge that different philosophical, theoretical, cultural or contextual orientations may influence the trustworthiness of the research. It is also important to note that the interviews took place before the COVID‐19 pandemic which is known to have had a significant impact on residents, staff and the wider nursing home carescape (Edvardsson et al., 2020). However, the concept of thriving‐promotion from the perspective of staff was previously unknown, so this study can provide insight into meaningful aspects that can be explored in contemporary care settings.

### 6.2 Recommendations for further research

Future research should explore the impact of interventions to support thriving and map thriving variability in relation to so‐called ‘traditional’ clinical variables, for example, frailty, falls, medication consumption and nutrition. As the literature on thriving perspectives is still emerging, it would be valuable to compare how staff perceive their role as co‐creators for resident thriving and the resident's own valuation of their thriving fulfilment. It could also be interesting to explore the extent to which promoting thriving results in personal fulfilment of nursing home staff. Furthermore, the role of managerial and organizational drivers should be considered in relation to staffs' ability to provide thriving‐promoting care. Given the undeniable impact of the pandemic on aged care delivery and routines, future studies could explore changes in the ways staff were, or were not, able to promote resident thriving before, during and after the pandemic, and the extent to which the restrictions impacted possibilities for health‐promoting interventions for residents.

### 6.3 Implications for policy and practice

Improving the health and well‐being of nursing home residents is an important nursing role. To promote resident thriving, nursing home staff could focus on targeted interventions relative to everyday care, activities, capabilities, relationships and the lived environment. The actions described herein may be further developed as practical health‐promoting clinical nursing interventions that highlight personalization, autonomy, connection and meaning‐making, and curation. It is important to develop education, clinical practice guidelines and documentation standards to better monitor and enhance thriving in nursing homes.

## 7 CONCLUSION

Nursing home staff promoted resident thriving by personalizing care and supporting autonomy, connection, meaning and pleasure. The findings provide practical descriptions and novel insights into how staff describe thriving promotion in nursing homes. Future research should further explore and quantify the extent to which staff education and care interventions are associated with improved resident thriving.

## AUTHOR CONTRIBUTIONS

All authors have agreed on the final version and meet at least one of the following criteria (recommended by the ICMJE*): (1) substantial contributions to conception and design, acquisition of data or analysis and interpretation of data; (2) drafting the article or revising it critically for important intellectual content. *https://www.icmje.org/recommendations/.

## FUNDING INFORMATION

This work was supported by The Swedish Research Council for Health, Working Life, and Welfare: FORTE [2014‐4016]; The Swedish Research Council [521‐2014‐2715]; and, The Medical Faculty, Umeå University [311‐839‐13].

## CONFLICT OF INTEREST STATEMENT

No conflict of interest has been declared by the authors.

### PEER REVIEW

The peer review history for this article is available at https://www.webofscience.com/api/gateway/wos/peer‐review/10.1111/jan.16206.

## Supporting information


Data S1:

## Data Availability

The data required to reproduce the study findings cannot be shared as the participants did not provide written consent for their raw data to be publicly distributed.
