# Supplementary material for: Promoting resident thriving in nursing homes: A qualitative study
Source: J Adv Nurs. 2024 Apr 25;81(1):399–408. doi: 10.1111/jan.16206 (PMC11638509; doi:10.1111/jan.16206)
Supplement: Supplementary file 1 — Data S1: [file JAN-81-399-s001.docx]

**Consolidated criteria for reporting qualitative studies (COREQ): 32-item checklist^[[1]](#footnote-1)^**

| **Item** | **CORE-Q checklist questions** | **Page** |
| --- | --- | --- |
| **Domain 1: Research team and reﬂexivity** | | |
| *Personal Characteristics* | | |
| 1. Interviewer/facilitator | Which author/s conducted the interview or focus group? | **7** |
| 2. Credentials | What were the researcher’s credentials? | **7,9** |
| 3. Occupation | What was their occupation at the time of the study? | **7** |
| 4. Gender | Was the researcher male or female? | **7** |
| 5. Experience and training | What experience or training did the researcher have? | **7** |
| *Relationship with participants* | | |
| 6. Relationship established | Was a relationship established prior to study commencement? | **7** |
| 7. Participant knowledge of the interviewer | What did the participants know about the researcher? | **7** |
| 8. Interviewer characteristics | What characteristics were reported about the interviewer/ facilitator? | **7,9** |
| **Domain 2: Study Design** | | |
| 9. Methodological orientation and Theory | What methodological orientation was stated to underpin the study? | **6,8** |
| *Participant selection* | | |
| 10. Sampling | How were participants selected? | **7** |
| 11. Method of approach | How were participants approached? | **7** |
| 12. Sample size | How many participants were in the study? | **8** |
| 13. Non-participation | How many people refused to participate or dropped out? Reasons? | **8** |
| *Setting* | | |
| 14. Setting of data collection | Where was the data collected? | **7** |
| 15. Presence of non-participants | Was anyone else present besides the participants and researchers? | **7** |
| 16. Description of sample | What are the important characteristics of the sample? | **9** |
| *Data collection* | | |
| 17. Interview guide | Were questions, prompts, guides provided by the authors? Was it pilot tested? | **7** |
| 18. Repeat interviews | Were repeat interviews carried out? If yes, how many? | **N/A** |
| 19. Audio/visual recording | Did the research use audio or visual recording to collect the data? | **7-8** |
| 20. Field notes | Were ﬁeld notes made during and/or after the interview or focus group? | **N/A** |
| 21. Duration | What was the duration of the interviews or focus group? | **8** |
| 22. Data saturation | Was data saturation discussed? | **8** |
| 23. Transcripts returned | Were transcripts returned to participants for comment and/or correction? | **N/A** |
| **Domain 3: analysis and ﬁndings** | | |
| *Data analysis* | | |
| 24. Number of data coders | How many data coders coded the data? | **8-9** |
| 25. Description of the coding tree | Did authors provide a description of the coding tree? | **8,  table 1** |
| 26. Derivation of themes | Were themes identiﬁed in advance or derived from the data? | **8** |
| 27. Software | What software, if applicable, was used to manage the data? | **N/A** |
| 28. Participant checking | Did participants provide feedback on the ﬁndings? | **N/A** |
| *Reporting* | | |
| 29. Quotations presented | Were participant quotations presented to illustrate the themes/ ﬁndings? Was each quotation identiﬁed? | **9-17** |
| 30. Data and ﬁndings consistent | Was there consistency between the data presented and the ﬁndings? | **9-17** |
| 31. Clarity of major themes | Were major themes clearly presented in the ﬁndings? | **9-17, table 2** |
| 32. Clarity of minor themes | Is there a description of diverse cases or discussion of minor themes? | **9-17** |

1. Developed from: Tong, A., Sainsbury, P., & Craig, J. (2007). Consolidated criteria for reporting qualitative research (COREQ): a 32-item checklist for interviews and focus groups. *International Journal for Quality in Health Care, 19*(6), 349 – 357. [↑](#footnote-ref-1)
